# Cyber violence caused by the disclosure of route information during the COVID-19 pandemic

**DOI:** 10.1057/s41599-022-01450-8

**Published:** 2022-11-25

**Authors:** Ying Lian, Yueting Zhou, Xueying Lian, Xuefan Dong

**Affiliations:** 1 School of Journalism, Communication University of China, No.1 Dingfuzhuang East Street, 100024 Beijing, People’s Republic of China; 2 College of Economics and Management, Beijing University of Technology, 100124 Beijing, People’s Republic of China; 3 Research Base of Beijing Modern Manufacturing Development, Beijing University of Technology, 100124 Beijing, People’s Republic of China

**Keywords:** Science, technology and society, Social policy, Cultural and media studies

## Abstract

Disclosure of patients’ travel route information by government departments has been an effective and indispensable pandemic prevention and control measure during the COVID-19 pandemic. However, this measure may make patients susceptible to cyber violence (CV). We selected 13 real cases that occurred in China during the COVID-19 pandemic for analysis. We identified several characteristics that commonly appeared due to route information, such as rumors about and moral condemnation of patients, and determined that patients who are the first locally confirmed cases of a particular wave of the pandemic are more likely to be the victims of CV. We then analyzed and compared six real cases using data mining and network analysis approaches. We found that disclosing travel route information increases the risk of exposing patients to CV, especially those who violate infection prevention regulations. In terms of disseminating information, we found that mainstream media and influential we-media play an essential role. Based on the findings, we summarized the formation mechanism of route information disclosure-caused CV and proposed three practical suggestions—namely, promote the publicity of the media field with the help of mainstream media and influential we-media, optimize the route information collection and disclosure system, and ease public anxiety about the COVID-19 pandemic. To our knowledge, this study is one of the first to focus on CV on social media during the COVID-19 pandemic. We believe that our findings can help governments better carry out pandemic prevention and control measures on a global scale.

## Introduction

### Cyber violence (CV)

Although the Internet has provided numerous advantages to the world, such as a convenient communication channel, rapid knowledge sharing, and a transparent public engagement process, some disadvantages have emerged, of which CV is a representative example. Definitions of CV vary but generally refer to internet-related behaviors that espouse violence or use language in a calculated manner to inflame passions and eventually achieve mass emotional catharsis (Peterson and Densley, 2017). According to several investigations, the prevalence of CV has reached a considerable level worldwide (Barlett et al., 2012; Cava et al., 2020; Jones et al., 2013; Peskin et al., 2017), especially among youths like students (Saleem et al., 2021). A national telephone survey of about 4500 youths aged 10–17 in 2000, 2005, and 2010 in the US reported a significant increase in the rate of online harassment between 2000 and 2010 (Jones et al., 2013). The survey also found that girls are more likely to experience CV, especially cyber dating violence. According to Hinduja et al., 20% of a random sample of 4441 students aged between 10 and 18 years experienced CV (Hinduja et al., 2013). In general, CV is much easier to perpetrate than traditional (or offline) violence because its carrier, the Internet, transcends geographic and temporal boundaries (Cava et al., 2020). However, CV can also generate the same adverse effects on victims as traditional violence, including but not limited to fear and other distressing emotions (Casas et al., 2013; Peterson and Densley, 2017) and even suicidal behavior. For example, a famous Korean actress committed suicide in October 2008 because of CV (Chiyohara, 2010).

### CV and social media

Many studies have demonstrated a relatively close relationship between CV and social media. For instance, Patton et al. (Patton et al., 2014) and Nagle (Nagle, 2018) systematically reviewed related studies and found significant positive correlations between CV and social media. Moreover, Ge found that CV frequently occurred on social networking sites like Twitter, Facebook, and Weibo (Ge, 2020). He further explained that users’ moral sensitivity could be reduced by social media, with CV constituting one of its main behavioral consequences. Ephraim found that CV against women and girls is becoming increasingly visible and prevalent, mainly contributing to increased social media use (Ephraim, 2013). Jane found that gendered CV on social media is becoming worse. Still, social media platform managers and policymakers fail to effectively address and prevent this problem, causing feminist digilantism to be more visible and forceful (Jane, 2017). Craig et al. conducted a cross-national study of 180,919 youths aged 11 to 15 from 42 countries to explore the relationship between cyberbullying and social media use (Craig et al., 2020). The results showed that the amount of time spent on social media was significantly correlated with the likelihood of being victimized by cyberbullying.

### CV in the COVID-19 pandemic

Since December 2019, COVID-19 has spread worldwide and has become an ongoing global health crisis. According to Tsao et al., as of January 2021, there had been more than 95 million cases of COVID-19, with about 2 million deaths (Tsao et al., 2021). In addition to its physical damage to human beings, COVID-19 has had adverse psychological impacts, of which CV has been the primary concern in recently published articles (Alsawalqa, 2021; Barlett et al., 2021; Barlett et al., 2021; Han et al., 2021; Jain et al., 2020; Li et al., 2020; Shirish et al., 2021; Yang, 2021). Most studies have posited a positive relationship between cyberbullying and the COVID-19 pandemic. For instance, Barlett et al. found that the prevalence of CV has significantly increased during the pandemic (Barlett et al., 2021). Han et al. proposed that the loneliness and isolation caused by the pandemic have stimulated CV behavior (Han et al., 2021). Yang conducted a web-based national survey of 5,608 individuals in China to investigate the correlation between CV, depression, and psychological coping strategies during the peak of the pandemic (Yang, 2021). The results showed that cyberbullying played a mediating role between the other two aspects.

There is no doubt that the government, as the leader and manager of society, should work to eliminate CV as much as possible, especially during major emergencies, such as the COVID-19 pandemic. However, one particular manifestation of CV during the pandemic has proven challenging to thwart—namely, route information disclosure-caused CV (RIDCCV). In this study, we defined RIDCCV as the CV caused by route information disclosure by the government. Generally, route information disclosure of infected patients is an effective tool for pandemic prevention and control, one which has been implemented by many countries, such as China and Japan. Route information disclosure is typically more frequent and common because national pandemic prevention and control measures tend to be normalized, and self-prevention consciousness can become fatigued (Morgul et al., 2021). However, according to previous studies, the personal information of infected individuals, including but not limited to their gender, age, place of residence, and recent travel route, is usually disclosed by the government through social media and official government websites or reposts that are available to the public to halt the spread of infection (Hu et al., 2020). These publication channels enable netizens to obtain the personal information of infected individuals much more quickly. They also create a platform that increases the risk of information leakage and CV to a large extent. Moreover, in most cases, grassroots staff in government departments are responsible for the route information disclosure of infected people if non-standard operations exist, such as unintentional dissemination of personal information, illegal sale of personal information, and insufficient removal of sensitive information before disclosure, information leakage, and CV would be easily triggered. Thus, although route information disclosure is of great significance in pandemic prevention and control, the probability of causing citizen information leakage and CV has increased.

Indeed, some existing studies have proposed that the information disclosure of infected people could lead them to suffer from abuse and discrimination during pandemics, such as COVID-19 (Deng and Feng, 2021; Di Trani et al., 2020; Jain et al., 2020; Liu et al., 2021). Liu et al. explained that this phenomenon is primarily caused by the perceived threat posed by infected individuals, which is a rapid and natural psychological response to emerging infectious diseases (Liu et al., 2021). In this view, the government can inadvertently stimulate CV. In addition, social media plays a promotional role in this process. In particular, social media allows people to post cyberbullying content with a low risk of exposing their identity (Vos et al., 2018). To date, few studies have focused on route information disclosure during the COVID-19 pandemic. Among them, Jung et al. (Jung et al., 2020) and Kim (Kim, 2021) focused on the privacy risks caused by the information disclosure practices of government departments in South Korea, in which route data were considered. However, they did not discuss any issues pertaining to CV.

The post-pandemic era is approaching, and the route information disclosure practices of governments are becoming increasingly normalized and frequent, which means that more RIDCCV may be aroused. There is no doubt that it is necessary to disclose the personal route information of confirmed patients and their close contacts to improve the effectiveness of epidemic prevention and control measures and interrupt the transmission of infectious diseases, such as COVID-19. Thus, all individuals have an obligation to cooperate with the epidemic prevention department by submitting the necessary aspects of their private lives. However, this does not mean unrestricted disclosure and dissemination of personal information. The excessive disclosure of personal information in the public sphere violates the right to privacy and affects everyday life (Mutimukwe et al., 2020), but it also negatively impacts the credibility of government agencies and hinders the normal progress of epidemic prevention and control (Jung et al., 2020).

Thus, it is of great significance to analyze real cases of RIDCCV and identify some valuable solutions to diminish or outright eliminate its occurrence, which has important implications for both current and future efforts to conduct better social governance during public emergencies, such as pandemics. We claim that, although reducing personal mobility is essential for preventing the spread of infectious diseases, each individual has the right to engage in outdoor activities without breaking the law. In addition, we assert that the anxiety caused by the COVID-19 pandemic is not a justification for CV.

This study aimed to address knowledge deficiencies concerning RIDCCV on social media during the COVID-19 pandemic. To achieve this goal, we first selected 13 representative cases of RIDCCV to identify typical characteristics. Six of these cases were then further analyzed. In particular, we collected and analyzed social media–based data about these six cases using data mining and network analysis approaches. The primary difference between the six cases is that three involved infected people who violated the Chinese epidemic prevention law. The other three were people who did not follow Chinese epidemic prevention recommendations. Specifically, this study sought to answer the following three research questions:

**RQ1:** What were the social media contents of the six cases of RIDCCV?

**RQ2:** Were there any differences between these six cases regarding the social media contents of RIDCCV?

**RQ3:** Who plays a vital role in disseminating information on social media?

The remainder of this paper is organized as follows. In section “Cases”, the 13 cases are described. Section “Methods” demonstrates the structure of the applied methods, including the data collection and mining approaches. Section “Results” presents the research results. Finally, section “Discussion and conclusions” presents our primary findings and discussions about the formation mechanism of RIDCCV in COVID-19 and proposes several practical recommendations.

## Cases

### Case 1

On December 8, 2020, the government of the Pidu District in Chengdu, Sichuan Province, officially announced that a woman, the granddaughter of two, was diagnosed with COVID-19. Her route information was also published, indicating that she had ventured out to many crowded public places before her diagnosis, including, but not limited to, a park and several bars. In addition, it should be noted that she was not required to isolate, meaning that she did not violate any epidemic prevention regulations. However, many netizens believed that her actions had placed them in danger. Thus, her personal information, such as her name, ID number, and family address, was exposed on the Internet. In the early morning of December 8, more people participated in the discussion of this event, the content of which was pervaded by a litany of negative opinions, such as speculation about the girl’s private life and insulting personal attacks.

### Case 2

A woman who returned from South Korea to Shenyang, Liaoning Province, on November 29, 2020, was asked to isolate herself at home. She was diagnosed with novel coronavirus pneumonia on December 23, 2020. On December 24, 2020, her route information following her return was announced by the Shenyang Health Commission, indicating that she did not fully comply with the quarantine regulations for inbound personnel in China. Specifically, during the isolation period, she went out to some crowded places despite having developed a fever. Because of her actions, 21 people were infected with COVID-19, and Shenyang began to carry out large-scale screening to detect potential infected cases. Thus, her personal information, such as her name, ID number, mobile phone number, and address, was exposed on the Internet. She and her family were cursed and abused by netizens on social media.

### Case 3

On August 4, 2021, government authorities disclosed the route information of a confirmed case of COVID-19, who had returned from Hainan Province to Beijing. According to previous media reports, the subject was a male teacher at the Central Academy of Fine Arts. They took China Southern Airlines flight CZ8804 from Sanya Airport to Beijing Daxing Airport on July 30. After he was diagnosed, the netizens speculated that he had gone to Sanya with his wife’s girlfriend and that she was also isolated. The rumor spread through the WeChat groups, resulting in CV against the man, which affected him, and the families involved.

### Case 4

On the evening of August 1, 2021, the Wuhan Economic Development Zone inspected the residents of a critical area through a tourist group in Huai’an, Jiangsu Province, and found that a migrant worker at a construction site had intersected with the activity itinerary of a tourist group in Huai’an while waiting for the train at the Jingzhou high-speed rail station on July 27. He underwent viral nucleic acid testing (NAT) on the morning of August 2, and the result was positive. His route information showed that he had traveled to many places in Wuhan on the 27th and 28th, and that two women were close contacts. Subsequently, netizens perpetuated rumors that the two women were the man’s “ex-girlfriend” and “current girlfriend,” and their personal information, such as ID numbers and telephone numbers, was exposed on the Internet. On August 5, the incident was further fermented, and negative opinions increased among netizens’ comments, such as speculation about private lives and other malicious remarks about the people involved.

### Case 5

On August 1, 2021, Shangqiu reported a new, locally confirmed COVID-19 case. The patient was hospitalized at Zhengzhou Sixth Hospital because her family was at high risk for COVID-19. The patient was the first in Shangqiu; after her diagnosis, she fully complied with the epidemic prevention and quarantine regulations. However, subsequently, her personal information was exposed on the Internet. She was rumored to be seriously ill or dead, and netizens further rumored that she had had an affair, which caused her online abuse that adversely affected her daily life.

### Case 6

A person who returned to Putian from Singapore was suspected to be the source of a wave of pandemic cases in Fujian. He returned on August 4, 2021, and fully complied with the epidemic prevention and control regulations during the quarantine period. Nine NAT results were negative, and one serum test was negative. He was diagnosed on September 10, but could not explain how he had become infected. After his route information was published on September 12, he received a large number of harassing phone calls, and some netizens spread rumors about his private life. This harassment seriously affected his daily life and made him worry about whether his family would also be affected.

### Case 7

On September 21, 2021, the Second People’s Hospital of Bayan County in Heilongjiang province reported a new case of a positive COVID-19 infection. The next day, the patient’s route information showed that she had taken the high-speed rail from Ji’an City to Nanchang and then flew from Nanchang to Harbin. After arriving in Harbin, she went to multiple restaurants and entertainment venues and played board games for three consecutive days. After her epidemiological investigation report was issued, her personal information, including her name, her partner’s name, and a detailed address, was exposed on the Internet. After that, the patient was accused by many netizens and even insulted online. In addition, she received moral criticism via CV because many netizens thought she should not have visited public places during the COVID-19 pandemic.

### Case 8

The government department disclosed the route information of two people with confirmed cases of COVID-19 who had returned from the Philippines to Harbin. They were two of the three first locally confirmed cases of a wave of the pandemic in Harbin. They had previously worked abroad and returned to China on August 3; after their return, they did not violate any epidemic prevention regulations, and their NAT results were negative. After the release of the quarantine on September 1, they went to multiple restaurants and entertainment venues. One of the persons was in close contact with the person described in Case 7 and had played live-action role laying (LARP) games with her. On September 21, Harbin Infectious Disease Hospital reported that the two were diagnosed as positive for COVID-19. After the government department disclosed their route information, the close contact of Case 7 suffered CV. His name and other personal information were leaked. Some netizens expressed dissatisfaction with his return to China, stating that they should keep away from crowded public places after their return during the COVID-19 pandemic.

### Case 9

On October 15, an elderly couple from Shanghai reportedly had abnormal NAT results in Jiayuguan City while traveling. However, they left for Xi’an alone after being informed not to leave. As of 17:30 on October 15, Jiayuguan City carried out an epidemiological investigation, NAT, personnel control, and environmental sanitizing at the places and with the contacts involved in the couple’s trip to detect potential infected cases. Then, on October 17, they were both confirmed to be infected, and the government disclosed their route information. CV occurred because some netizens thought they should not have left for Xi’an alone. They were condemned and targeted with personal abuse online. However, on October 18, the reporter covering the case interviewed the couple and the Gansu Provincial Health Commission staff exclusively and found that the statement “left by themselves” was untrue. The couple did not travel without authorization after testing positive and did not violate epidemic prevention and control regulations during the period. After this revelation, some netizens expressed sympathy for the two elderly people. However, other netizens noticed that when the two were in Xi’an, after learning that their NAT results were abnormal and before the NAT retest results came out, they still went to other places in Xi’an. Therefore, some netizens condemned this behavior, and at the same time, there were unfavorable comments on regional epidemic prevention and control.

### Case 10

A male in Wulian County, Rizhao, Shandong Province, tested positive for COVID-19 via NAT on the evening of October 25 and had a history of living in Yinchuan City, Ningxia, from October 15 to 18. On October 26, Wulian County officials held a press conference on COVID-19. After the subject had arrived in Rizhao on the 18th, he tested negative on the 19th, and then he was active mainly near his work unit and residential area. On October 25, he went to the rehabilitation hospital and was later diagnosed. After the Chinese Center for Disease Control and Prevention announced the case, some netizens said that he had concealed the trip. However, the official did not confirm the matter. His ID and telephone numbers were leaked, and he was attacked by public opinion.

### Case 11

On October 10, a tour guide took a team to the Ceke port (where there had been a previous epidemic) for a short visit. The head of the travel agency where the guide worked said that after ending the trip to Ejina Banner, Inner Mongolia, the tour guide was infected with COVID-19 while taking another team in Gannan Prefecture, Gansu Province. The tour group colleagues and tourists in close contact with him tested negative. After the tour guide was diagnosed, his information was leaked, and he was targeted with personal abuse online.

### Case 12

On October 13, a male subject returned to Shijiazhuang from Ejina Banner, Inner Mongolia, where the epidemic already existed. He had lived and worked in Ejin Naqi from September 26 to October 12, but he did not report his travel as soon as he returned. On October 22, after the man learned that people with a history of travel in medium- and high-risk areas needed to report, he took the initiative to report his trip without concealing his residence history. On November 1, he was notified of a positive COVID-19 diagnosis. After his route information was disclosed, he was condemned and received personal abuse online because his failure to report in time triggered small disease outbreaks in Shenze, Xinji, and other places.

### Case 13

On November 1, the Fourth People’s Hospital of Chengdu reported that a male subject had a positive NAT for the new coronavirus. The Municipal Center for Disease Control and Prevention confirmed this case. The man traveled to Xi’an, Yinchuan, Lanzhou, and Chongqing on a business trip from October 8 to 27 and drove back to Chengdu on the 28th. During the business trip, he had symptoms such as dizziness and chills, but after visiting the doctor on the 27th, he obtained a negative NAT report. He did not report to the community after his return on the 28th. The next day, he developed fever symptoms, but he denied any travel history in medium- and high-risk areas in the country at the time of treatment. After his route information was disclosed, he became the target of CV because of violations of epidemic prevention and control regulations. He was accused, insulted, and personally attacked by netizens.

## Methods

### Data collection

First, we identified 13 representative cases of RIDCCV that occurred between November 2020 and November 2021 in China by devoting continuous attention to online posts related to RIDCCV on social media. Then, we employed three doctoral students with research experience in CV to ascertain the whole story of each case by manually searching relevant information on the Internet and manually confirming whether the following six features existed:

- The person violated epidemic prevention and control regulations.
- The person was the first locally confirmed case of a wave of the pandemic.
- Personal information was leaked (name, telephone, etc.).
- The person was nicknamed by people on social media.
- There were rumors.
- There was moral criticism.

Six cases were then considered for further analysis: Case 1, Case 2, Case 4, Case 7, Case 9, and Case 13. We chose these six cases for analysis because they generated more online posts than the other cases. In addition, they can be classified into two markedly different groups: people who had violated epidemic prevention and control regulations (Case 2, Case 9, and Case 13) and people who did not (Case 1, Case 4, and Case 7). This difference could, to a large extent, improve the richness of the data we collected from social media. In other words, conducting a comprehensive comparative analysis based on public opinions on social media concerning these cases was possible. Moreover, to eliminate the ambiguity caused by their names, we renamed Case 1, Case 2, Case 4, Case 7, Case 9, and Case 13 as Research Case 1 (RCase 1), Research Case 2 (RCase 2), Research Case 3 (RCase 3), Research Case 4 (RCase 4), Research Case 5 (RCase 5), and Research Case 6 (RCase 6), respectively.

The social media platform Weibo (Chinese Twitter) was utilized to obtain data regarding the RIDCCV on the six cases during the COVID-19 pandemic. According to Li et al. (2020), Weibo has been one of the leading platforms employed by the Chinese government to disclose COVID-19-related information. Many Chinese government agencies have created their own official accounts on Weibo. Weibo has been deployed as a data source by many studies focusing on issues in China (Tao and Su, 2021; Wu et al., 2021). Octopus, a mature web crawler tool, was used to collect data from Weibo, as it has shown great effectiveness in previous studies (Li et al., 2021; Liu and Hu, 2019). In addition, it should be noted that the data collection process was implemented daily to acquire the maximum amount of data. The data were collected as follows:

- For RCase 1, we collected the original posts containing the keywords “girl,” “COVID-19,” and “Pixian County” on Weibo and their associated comments from December 7 to December 16, 2020, yielding a total of 63,719 online posts.
- For RCase 2, we collected original posts containing the keywords “old lady Yin,” “COVID-19,” and “Shenyang” on Weibo and their associated comments from December 23, 2020 to January 18, 2021, which yielded a total of 47,913 online posts.
- For RCase 3, original posts containing the keywords “Wuhan” and “COVID-19” on Weibo and their associated comments from August 5 to 11, 2021 were collected, which yielded a total of 13,840 online posts.
- For RCase 4, the applied keywords were “Harbin,” “COVID-19,” and “LARP Games,” and the collection period was between September 21 and September 29, 2021, which yielded 17,037 online posts.
- For RCase 5, we collected original posts containing the keywords “Xi’an” and “COVID-19” on Weibo and their associated comments from October 17 to 24, 2021, which yielded 21,896 online posts.
- For RCase 6, original posts containing the keywords “Chengdu” and “COVID-19” on Weibo and their associated comments from November 5 to 12, 2021 were collected, yielding a total of 13,114 online posts.

The collected data included user names, posting times, and the content of the posts. We then used the data-cleaning function of the Statistical Package for the Social Sciences (SPSS) version 25.0 (IBM Corp., Armonk, NY) to delete incomplete data. Invalid data, such as advertisements, were manually removed. Finally, the amount of remaining data related to the six cases comprised 62,888, 45,538, 12,558, 15817, 21,073, and 12,291 items, respectively.

### Data mining methods

To answer the first research question—*What were the social media content of the six cases of RIDCCV?—*text mining analysis was performed on collected online posts related to six RCases via natural-language processing techniques. The following information demonstrates the methods used in this research.

#### Sentiment analysis

Sentiment analysis was applied to assess how the public felt about the topic. In this study, we employed bi-directional long short-term memory (Bi-LSTM), a commonly applied deep learning algorithm in sentiment analysis. Bi-LSTM is composed of a forward LSTM and a backward LSTM and has demonstrated optimal performance in processing Chinese microblogging data for sentiment analysis (Dou et al., 2021; Ling et al., 2020). In addition, the R package of Jieba with the basic lexicon of Sougou Pinyin was used to finish the word segmentation task. Word2Vec, a word vector training tool launched by Google, was used for word embedding. For the Bi-LSTM, the hidden size of each LSTM unit was set at 300, and the learning rate was set at 0.01 for optimization. We used a dataset containing 120,000 online posts collected from Weibo to train the model, half labeled as positive and half labeled as negative. These data are available at https://qcsdn.com/q/a/49489.html. We then used the obtained classifier to identify the sentiment tendencies in our collected online posts.

To test the overall performance of the results, three students were employed to manually check the accuracy of the sentiment classification of 5,000 online posts. Only those considered correct by all three students were classified in the correct group. The final average accuracy rate of the 5,000 tested data for each RCase was 89.77%, indicating an acceptable margin of error. Thus, the sentiment tendency results were highly reliable.

#### Topic clustering

Many topic clustering algorithms have been proposed by existing studies, of which Latent Dirichlet Allocation (LDA) is one of the most commonly applied models. LDA has been employed in various areas, such as politics, technology, economy, management, and transportation. Inspired by these studies, this study used LDA to implement text analysis for topic clustering regarding the collected data. The basis of LDA refers to the statistical correlation of words presented in researched documents without considering word order. For parameter settings, the k-dimensional topic smoothing parameter and the k-dimensional word smoothing parameter are set as 0.1 and 0.01, respectively (Xu et al., 2018). Additionally, the trial-and-error method was used to determine the optimum value of the number of topics. According to Kaplan and Vakili (2015), although this method spends much time repeating the clustering procedures with varying numbers of topics, its accuracy can be guaranteed to a large extent. Before carrying out topic clustering, we used Jieba to segment the collected posts by words. The term frequency-inverse document frequency (TF-IDG) method was applied to measure the weight of each characteristic word in each post. TF-IDF is a numerical statistic method for assessing the importance of each word in particular documents, which has been commonly applied by previous studies in information retrieval and (Kim et al., 2019; Mee et al., 2021). The calculation method of TF-IDF is displayed as formula (1).

1 $$W_{ij} = {\mathrm{TF}}_{ij} \times {\mathrm{ID}}_{ij} = {\mathrm{TF}}_{ij} \times \log (N/{\mathrm{DF}}_j)$$

In formula (1), *W*_*ij*_, TF_*ij*,_ and ID_*ij*_ represent the weight, word frequency, and reverse file frequency of word *i* in document *j*, respectively. *N* is the total number of documents, and DF_*j*_ is the total number of documents containing the word *i*.

### Social network analysis

To investigate the role played by different users in disseminating information related to RIDCCV on social media, the social network analysis method was applied. In particular, based on the collected data, we constructed the network spread of the six cases based on the original posts and comments. Each node represents a user, and each edge measures a commenting relation. The constructed networks were both weighted-directed networks, in which the weight was measured as the number of commenting relations. Then, we carried out a weighted PageRank analysis using the method proposed by Zhang to further explore the importance of each user in the spreading networks (Zhang, 2017).

In addition, to provide a more comprehensive and detailed picture of the user, we conducted a classification analysis. According to existing studies focusing on information spread, mainstream media and we-media were commonly considered and compared (Luqiu et al., 2019; Wang, 2021; Wu, 2018; Xu et al., 2018). Mainstream media is the media that has an enormous influence, reaches a large mainstream audience, guides public opinion, and generates a strong social influence. In comparison, we-media is a new type of media formed based on the development of microblog technology. In general, there are two different arguments regarding the differences between mainstream media and we-media. One is that mainstream media equals traditional media, such as online news, television, and major websites, while we-media equals microblogs, such as Twitter and Weibo (Peng et al., 2015; Wang, 2021). The other argument is that mainstream media equals official media, while we-media can be viewed as non-official media created by individuals or non-official organizations (Al-Zaman and Noman, 2021; Wu, 2018). In this study, the latter classification criteria were employed. Furthermore, following Dong and Lian (2022), we used the industrial category, an attribute of user profiles on Weibo, to determine whether a given user was official or not. In particular, Dong and Lian (2022) identified 70 official categories, such as government–emergency management, newspaper–official newspaper, government–technology, and government–judicial administration.

We also considered the influence of we-media based on their number of followers. Users were classified into four groups according to the profiles displayed on the homepage of each user on Weibo: mainstream/official media, influential media, we-media, and normal users. We-media users who had more than 300,000 followers were classified into the influential we-media group. Until now, there have been no accurate criteria for judging whether a user was an influential user. Although the number of followers proved to be an essential indicator in many previous studies (Ge, 2020; Luqiu et al., 2019; Shi et al., 2021), the aim of this study was not to accurately identify influential users but rather to provide a relatively detailed understanding of the role of we-media in spreading information on social media. Thus, our classification criteria were largely rational.

## Results

### Characteristics of 13 RIDCCV cases

The coding results regarding the characteristics of the 13 selected RIDCCV cases are shown in Table 1, with the summarized results shown in Tables 2 and 3. The CV victims in 11 of the cases represent the first locally confirmed case of a wave of the pandemic, and personal information was leaked (11 of 13 cases). In addition, some cases demonstrated the existence of moral criticism and rumors caused by disclosing the subject’s travel route information.

**Table 1:** 13 RIDCCV cases that occurred in China between November, 2020 and November, 2021.

	Place	Time	Characteristics	
1	Chengdu, Sichuan Province	8-Dec-20	Whether the person violated epidemic prevention and control regulations	No
			Whether the person was the first locally confirmed case of a wave of the pandemic	Yes
			Whether there was personal information leakage (name, telephone, etc.)	Yes
			Whether the person was nicknamed by people on social media	Yes
			Whether there were rumors	Yes
			Whether there was moral criticism	Yes
2	Shenyang, Liaoning Province	23-Dec-20	Whether the person violated epidemic prevention and control regulations	Yes
			Whether the person was the first locally confirmed case of a wave of the pandemic	Yes
			Whether there was personal information leakage (name, telephone, etc.)	Yes
			Whether the person was nicknamed by people on social media	Yes
			Whether there were rumors	Yes
			Whether there was moral criticism	Yes
3	Beijing	4-Aug-21	Whether the person violated epidemic prevention and control regulations	No
			Whether the person was the first locally confirmed case of a wave of the pandemic	No
			Whether there was personal information leakage (name, telephone, etc.)	No
			Whether the person was nicknamed by people on social media	No
			Whether there were rumors	Yes
			Whether there was moral criticism	Yes
4	Wuhan, Hubei Province	5-Aug-21	Whether the person violated epidemic prevention and control regulations	No
			Whether the person was the first locally confirmed case of a wave of the pandemic	Yes
			Whether there was personal information leakage (name, telephone, etc.)	Yes
			Whether the person was nicknamed by people on social media	Yes
			Whether there were rumors	Yes
			Whether there was moral criticism	Yes
5	Shangqiu, Henan Province	15-Aug-21	Whether the person violated epidemic prevention and control regulations	No
			Whether the person was the first locally confirmed case of a wave of the pandemic	Yes
			Whether there was personal information leakage (name, telephone, etc.)	Yes
			Whether the person was nicknamed by people on social media	No
			Whether there were rumors	No
			Whether there was moral criticism	No
6	Putian, Fujian Province	12-Sep-21	Whether the person violated epidemic prevention and control regulations	No
			Whether the person was the first locally confirmed case of a wave of the pandemic	Yes
			Whether there was personal information leakage (name, telephone, etc.)	Yes
			Whether the person was nicknamed by people on social media	No
			Whether there were rumors	No
			Whether there was moral criticism	No
7	Heilongjiang, Harbin Province	21-Sep-21	Whether the person violated epidemic prevention and control regulations	No
			Whether the person was the first locally confirmed case of a wave of the pandemic	Yes
			Whether there was personal information leakage (name, telephone, etc.)	Yes
			Whether the person was nicknamed by people on social media	No
			Whether there were rumors	No
			Whether there was moral criticism	Yes
8	Heilongjiang, Harbin Province	21-Sep-21	Whether the person violated epidemic prevention and control regulations	No
			Whether the person was the first locally confirmed case of a wave of the pandemic	Yes
			Whether there was personal information leakage (name, telephone, etc.)	Yes
			Whether the person was nicknamed by people on social media	No
			Whether there were rumors	No
			Whether there was moral criticism	No
9	Xi’an, Shaanxi Province	17-Oct-21	Whether the person violated epidemic prevention and control regulations	Yes
			Whether the person was the first locally confirmed case of a wave of the pandemic	Yes
			Whether there was personal information leakage (name, telephone, etc.)	Yes
			Whether the person was nicknamed by people on social media	No
			Whether there were rumors	Yes
			Whether there was moral criticism	No
10	Rizhao, Shandong Province	25-Oct-21	Whether the person violated epidemic prevention and control regulations	No
			Whether the person was the first locally confirmed case of a wave of the pandemic	Yes
			Whether there was personal information leakage (name, telephone, etc.)	Yes
			Whether the person was nicknamed by people on social media	No
			Whether there were rumors	No
			Whether there was moral criticism	No
11	Gannan, Gansu Province	27-Oct-21	Whether the person violated epidemic prevention and control regulations	Yes
			Whether the person was the first locally confirmed case of a wave of the pandemic	No
			Whether there was personal information leakage (name, telephone, etc.)	Yes
			Whether the person was nicknamed by people on social media	No
			Whether there were rumors	No
			Whether there was moral criticism	No
12	Shijiazhuang, Hebei Province	4-Nov-21	Whether the person violated epidemic prevention and control regulations	No
			Whether the person was the first locally confirmed case of a wave of the pandemic	Yes
			Whether there was personal information leakage (name, telephone, etc.)	No
			Whether the person was nicknamed by people on social media	Yes
			Whether there were rumors	No
			Whether there was moral criticism	No
13	Chengdu, Sichuan Province	5-Nov-21	Whether the person violated epidemic prevention and control regulations	Yes
			Whether the person was the first locally confirmed case of a wave of the pandemic	Yes
			Whether there was personal information leakage (name, telephone, etc.)	Yes
			Whether the person was nicknamed by people on social media	No
			Whether there were rumors	No
			Whether there was moral criticism	No

**Table 2:** Summarized results of characteristics of 13 RIDCCV cases.

Characteristics	Yes	No
Whether the person violated epidemic prevention and control regulations	4	9
Whether the person was the first locally confirmed case of a wave of the pandemic	11	2
Whether there was personal information leakage (name, telephone, etc.)	11	2
Whether the person was nicknamed by people on social media	4	9
Whether there were rumors	5	8

**Table 3:** Three main topics extracted from the data focusing on both RCase 1 and RCase 2.

	Topics	Examples
1	Hope the epidemic will end soon and call on the government to find out the source of the epidemic as soon as possible.	“*Hope Chengdu and Shenyang will return to zero confirmed cases as soon as possible*.” “*It is hoped that the government can find out the infection source of the outbreak in Pidu district in Chengdu*.”
2	Call on the government to strengthen management in route information disclosure.	“*Just after the case occurred in Chengdu, the case in Shenyang leaked people’s personal information again. We should find out the person and unit responsible for this mistake*.” “*If patient’s information is not effectively kept confidential, suspected patients will hide their movements and will not actively cooperate with the treatment. This is extremely unfavorable to epidemic control! The government should protect the patient’s privacy. It happened once in Chengdu and once in Shenyang. Similar privacy leakage can no longer occur. The person responsible should be investigated and affixed*.”
3	Sympathize with the girl in RCase 1 and think the woman in RCase 2 deserves cyber violence and is not worth being pitied.	“*This case really makes people angry, which is completely different from the nature of the case occurred in Chengdu. Support investigating the criminal accountability of the woman*.” “*Compared with the girl in Chengdu, this woman should be scolded. The girl reported the route information at the first time of diagnosis, while the woman lied about the track. She is really detestable*.”

### Text mining results for six RCases

#### RCase 1

**Fig. 1:**
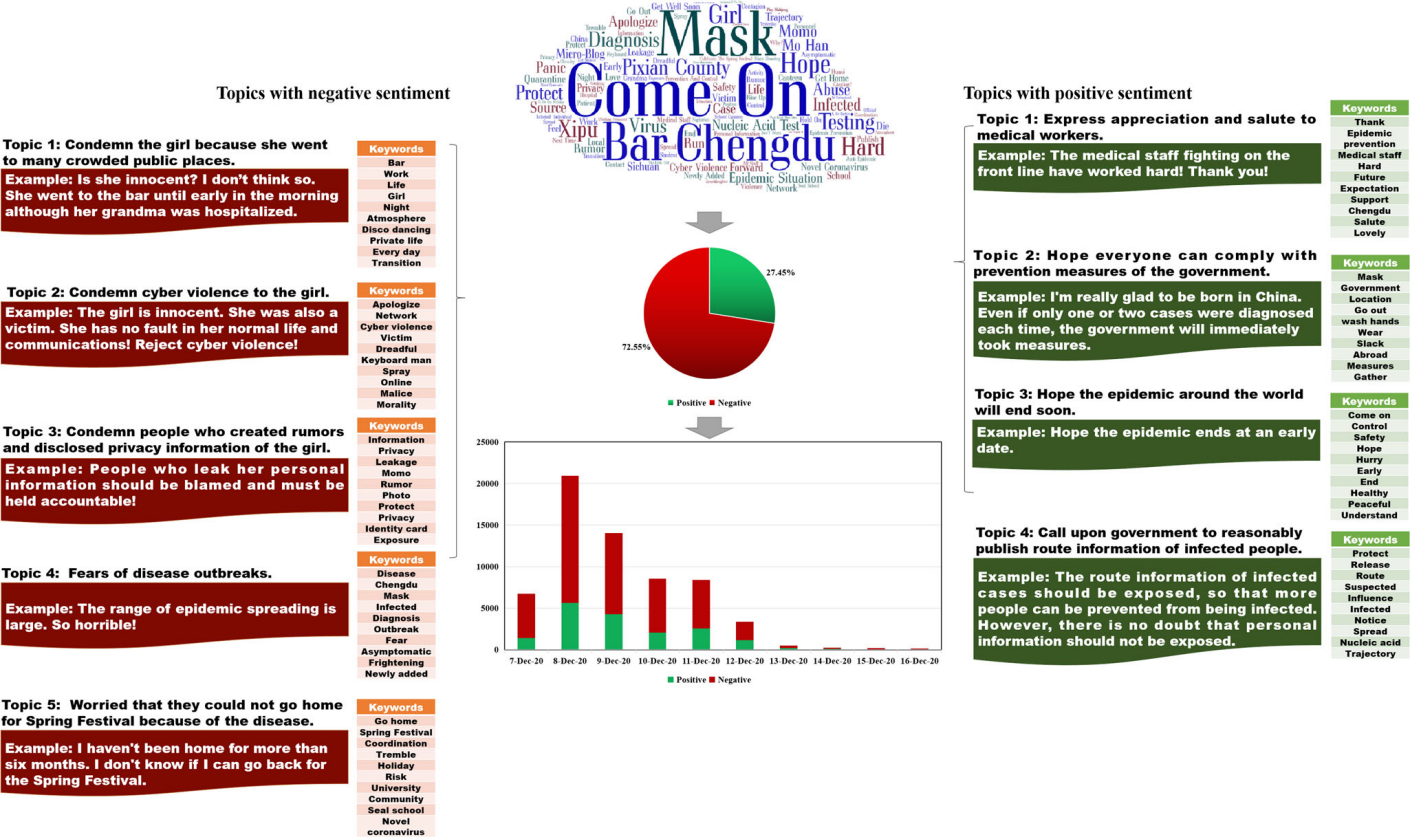
Content analysis results of RCase 1. This figure shows the sentiment analysis and topic clustering results of RCase 1.

Figure 1 displays the content analysis results of RCase 1. We extracted the top 100 keywords based on the ranking results of the TF-IDF values. The figure shows that some general words, such as “Come on” and “Chengdu,” also had high TF-IDF values. In addition, it is interesting to note that “Bar” had a considerably high TF-IDF value, which reflects how people seemed to pay more attention to the fact that the girl in RCase 1 visited several bars. Indeed, debates about whether “Should the girl go to bars?” represented a new derived topic in RCase 1. These debates occurred partly because, in traditional Chinese conceptions, young girls visiting bars is discouraged, although the younger generation in China generally disagrees with this view. For instance, someone asked, “Why do so many people think going to bars means bad children?” Regarding the sentiment analysis results, the proportion of negative and positive comments was 72.55% and 27.45%, respectively. Five topics with a negative tendency and four with a positive tendency were extracted from the commenting data. Specifically, for the topics with a negative sentiment tendency, “Condemn the girl because she went to many crowded public places,” “Condemn cyber violence to the girl,” “Condemn people who created rumors and disclosed privacy information of the girl,” “Fears of disease outbreaks,” and “Worried that they could not go home for Spring Festival because of the disease” were obtained. In terms of topics with a positive sentiment tendency, “Express appreciation and salute to medical workers,” “Hope everyone can comply with prevention measures of the government,” “Hope the epidemic around the world will end soon,” and “Call upon government to reasonably publish route information of infected people” were identified.

#### RCase 2

**Fig. 2:**
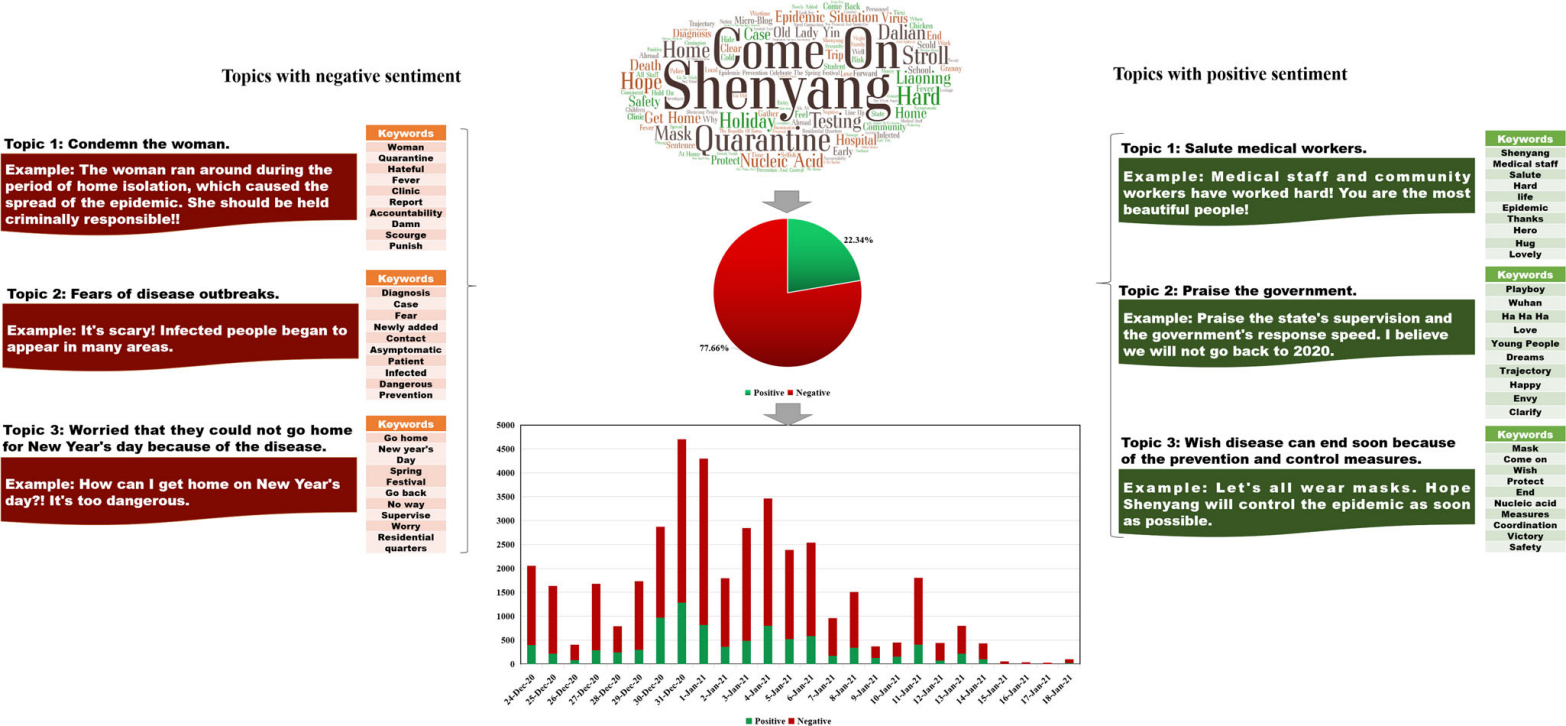
Content analysis results of RCase 2. This figure shows the sentiment analysis and topic clustering results of RCase 2.

Figure 2 displays the content analysis results of RCase 2. Here, we also extracted the top 100 keywords according to the ranking results of the TF-IDF values. Similar to the results of Case 1, in addition to some general words, such as “Come on” and “Shenyang,” keywords with high TF-IDF values also included “Quarantine,” “Stoll,” “Home,” and “Hard.” This finding indicates that most people were concerned about the traveling behavior of the woman during quarantine. Concerning the sentiment analysis results, it can be noted that the proportion of negative and positive comments was 77.66% and 22.34%, respectively. Three topics with a negative tendency and three with a positive tendency were extracted from the commenting data. Specifically, for the topics with a negative sentiment tendency, “Condemn the woman,” “Fears of disease outbreaks,” and “Worried that they could not go home for New Year’s Day because of the disease” were obtained. In terms of topics with a positive sentiment tendency, “Salute medical workers,” “Praise the government,” and “Wish disease can end soon because of the prevention and control measures” were identified.

#### RCase 3

**Fig. 3:**
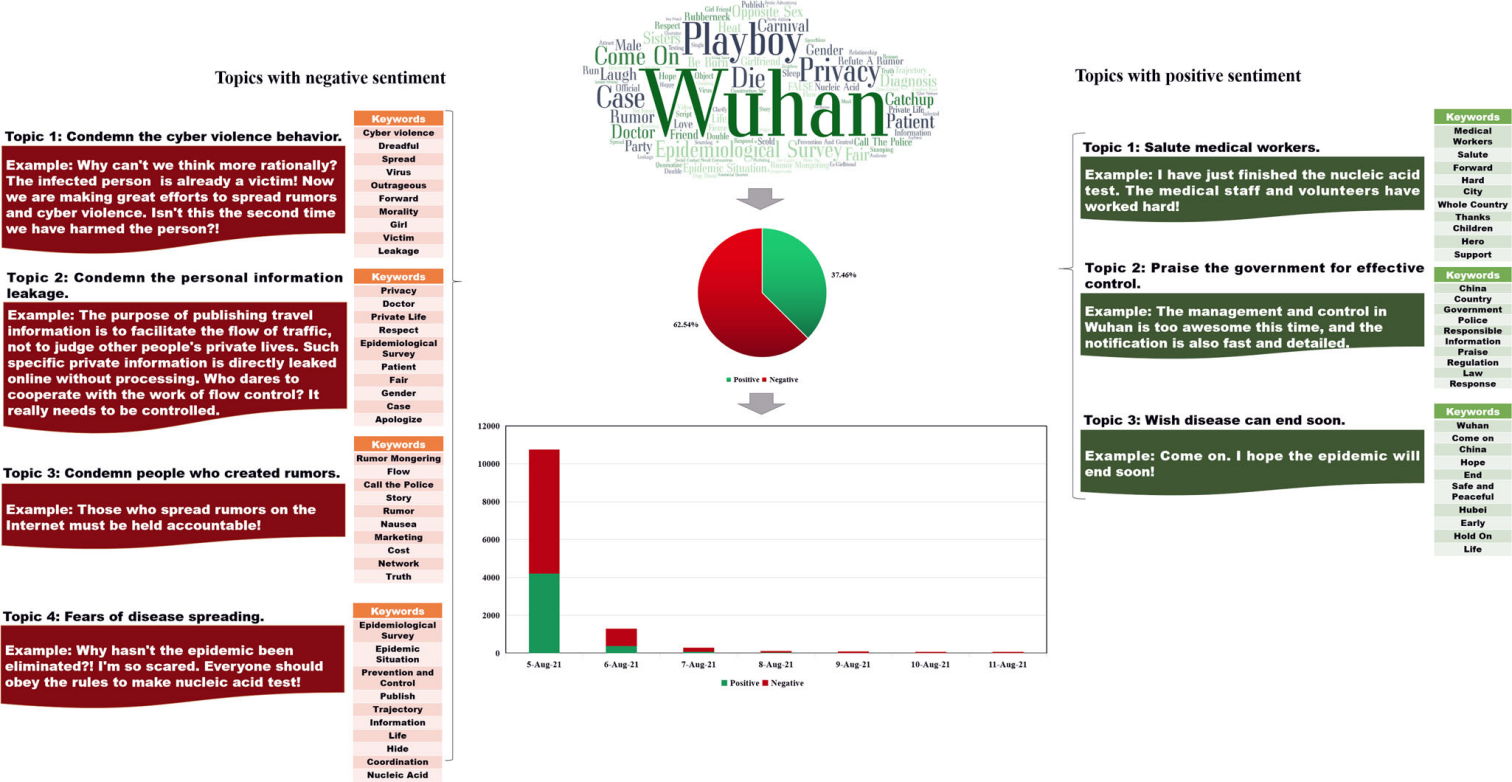
Content analysis results of RCase 3. This figure shows the sentiment analysis and topic clustering results of RCase 3.

Figure 3 displays the content analysis results of RCase 3. Based on the TF-IDF values, we identified the top 100 keywords. In addition to some general words, such as “Wuhan” and “Come on,” the keywords “Playboy” (which relates to the specific story of this case), “Privacy,” and “Epidemiological Survey” (which are related to the leaks of personal information in the epidemiological survey announcement) had high TF-IDF values as well. Regarding the sentiment analysis results, the proportion of negative and positive comments was 62.54% and 37.46%, respectively. Moreover, a rapidly decreasing trend can be found in the number of online posts related to this case. For the topic clustering results, four topics with a negative tendency were identified: “Condemn the cyberviolence behavior,” “Condemn the personal information leakage,” “Condemn people who created rumors,” and “Fears of disease spreading.” In addition, three topics with a positive tendency were obtained, including “Salute medical workers,” “Praise the government for effective control,” and “Wish disease can end soon.”

#### RCase 4

**Fig. 4:**
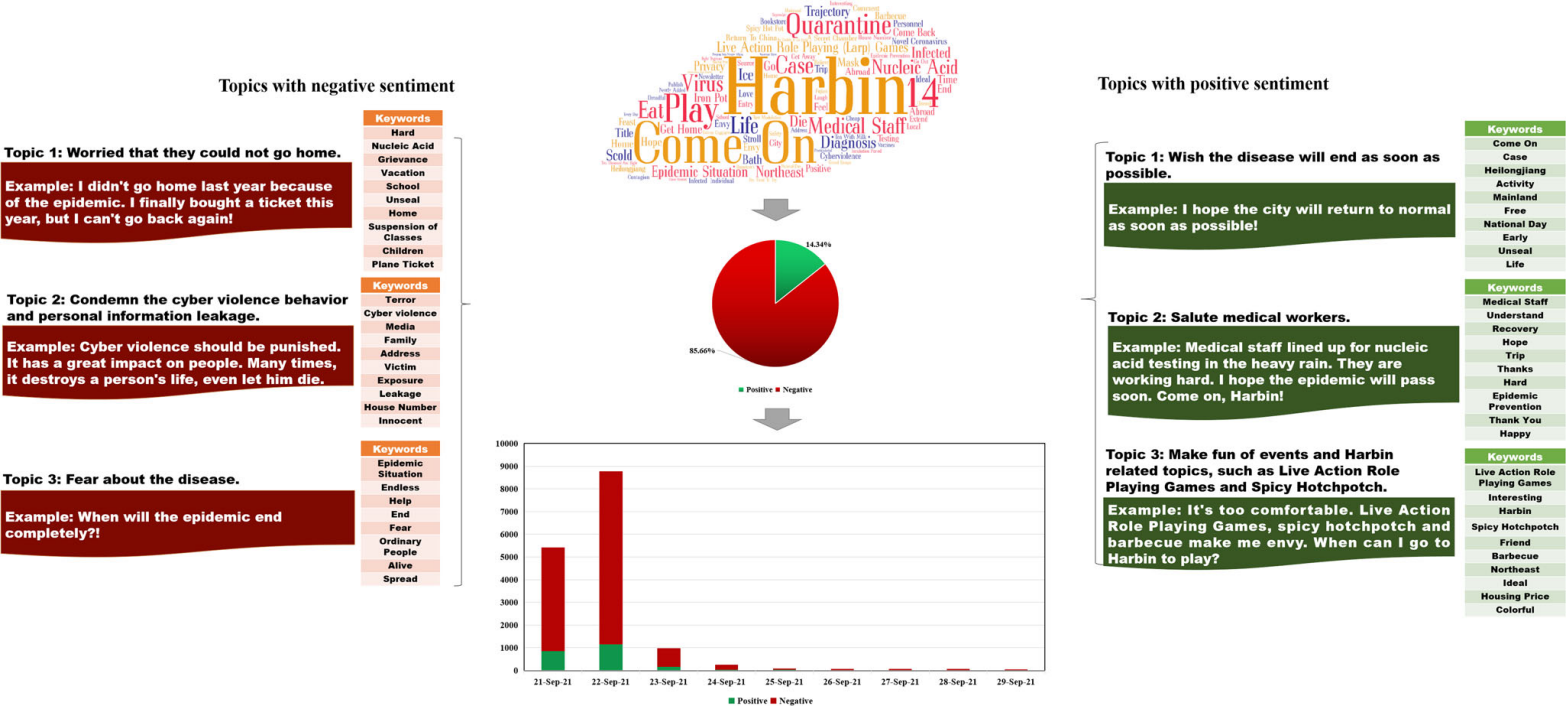
Content analysis results of RCase 4. This figure shows the sentiment analysis and topic clustering results of RCase 4.

Figure 4 displays the content analysis results of RCase 4. We extracted the top 100 keywords according to the ranking results of the TF-IDF values, which shows that the keywords “Play,” “Quarantine,” “LARP Games,” and “Nucleic Acid” had high TF-IDF values. This finding indicates that most people were concerned that the infected person played LARP games for three consecutive days in Harbin, which caused many infections. Concerning the sentiment analysis results, it can be noted that the proportion of negative and positive comments was 85.66% and 14.34%, respectively. Three topics with a negative tendency and three with a positive tendency were extracted from the commenting data. In particular, for the topics with a negative sentiment tendency, “Worried that they could not go home,” “Condemn the cyberviolence behavior and personal information leakage,” and “Fear about the disease” were obtained. These topics are similar to those obtained in RCase 1 because both cases had similar timings, occurring close to holidays. In terms of topics with a positive sentiment tendency, we identified “Wish the disease will end as soon as possible,” “Salute medical workers,” and “Make fun of events and Harbin-related topics, such as LARP games and Spicy Hotchpotch.”

#### RCase 5

**Fig. 5:**
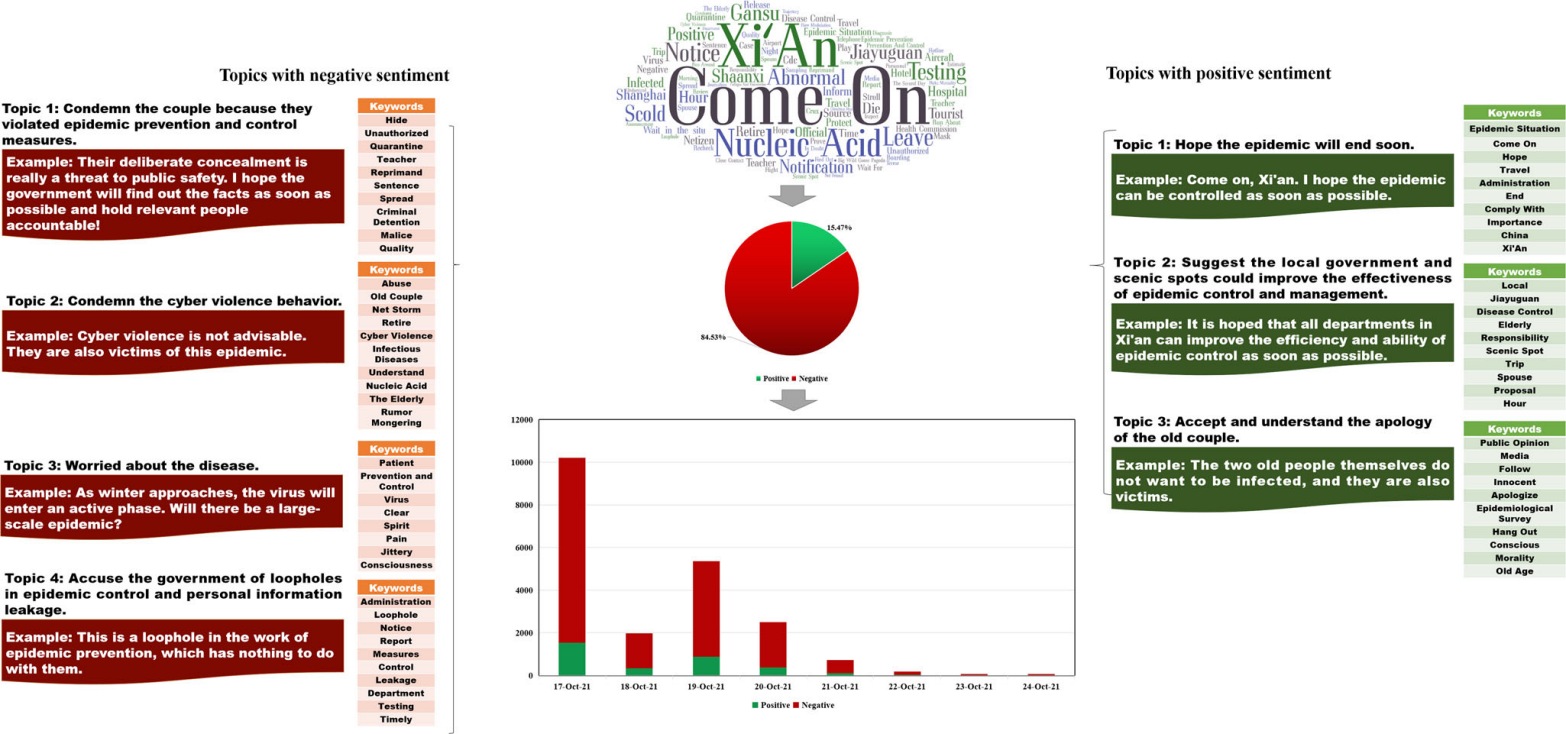
Content analysis results of RCase 5. This figure shows the sentiment analysis and topic clustering results of RCase 5.

Figure 5 displays the content analysis results of RCase 5. The results regarding the top 100 keywords based on the TF-IDF values showed that except for some general keywords such as “Come On” and “Xi’An,” the keywords “Jiayuguan” (a famous scenic spot in Xi’an, China), “Nucleic Acid,” “Gansu,” “Abnormal,” and “Scold” had high TF-IDF values. Regarding the sentiment analysis results, 84.53% of the collected comments showed a negative sentiment tendency, and 15.47% showed a positive tendency. In addition, four negative topics were identified: “Condemn the couple because they violated epidemic prevention and control measures,” “Condemn the cyber violence behavior,” “Worried about the disease,” and “Accuse the government of loopholes in epidemic control and personal information leakage.” The topics with a positive sentiment tendency were “Hope the epidemic will end soon,” “Suggest the local government and scenic spots could improve the effectiveness of epidemic control and management,” and “Accept and understand the apology of the old couple.”

#### RCase 6

**Fig. 6:**
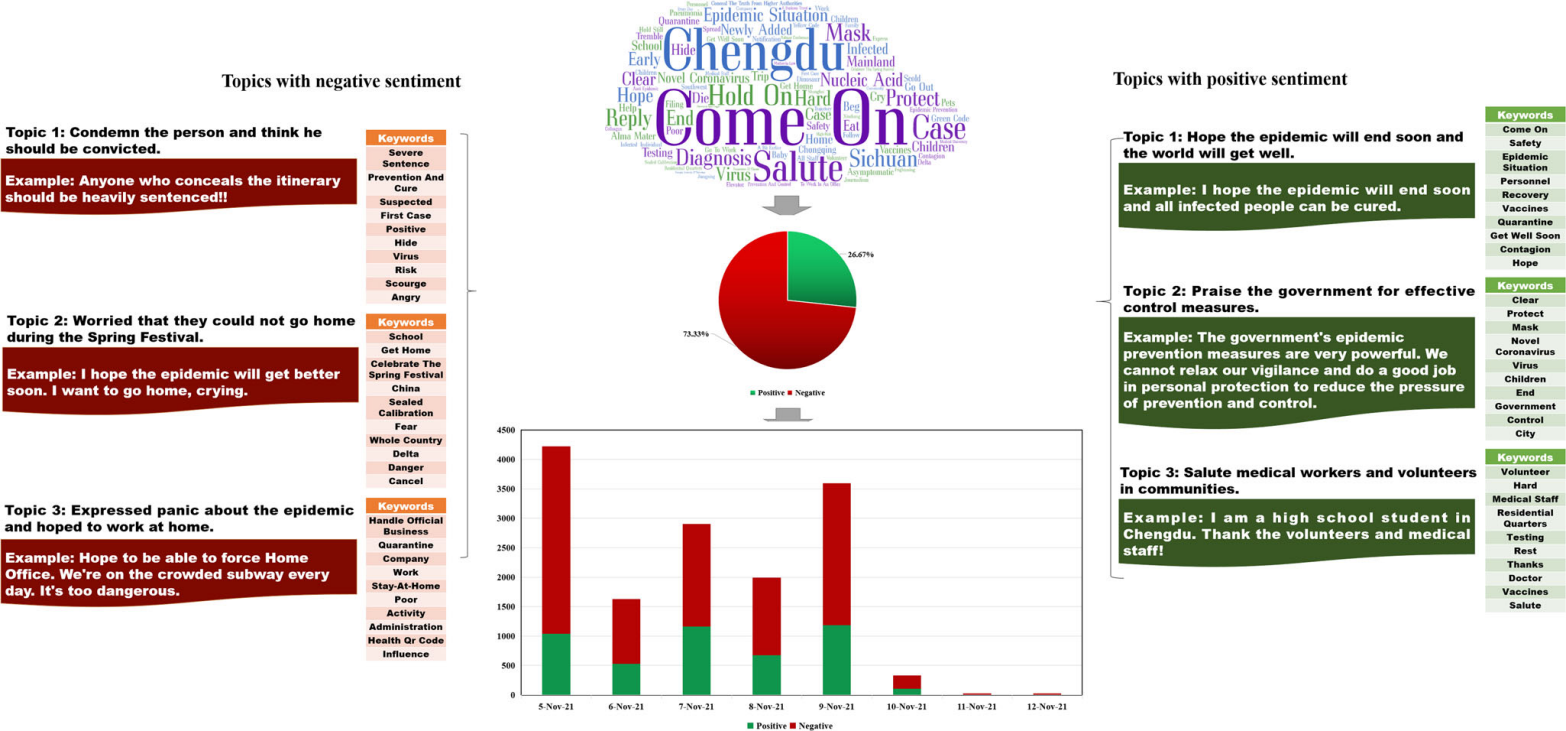
Content analysis results of RCase 6. This figure shows the sentiment analysis and topic clustering results of RCase 6.

Figure 6 displays the content analysis results of RCase 6. According to the results, the keywords related to the place of occurrence and epidemic prevention and control have high TF-IDF values, including “Chengdu,” “Come On,” “Salute,” “Diagnosis,” “Hold On,” and “Mask.” For the sentiment analysis results, the proportion of negative and positive comments was 73.33% and 26.67%, respectively. In addition, three topics with a negative tendency and three with a positive tendency were extracted from the commenting data. Specifically, for the topics with a negative sentiment tendency, “Condemn the person and think he should be convicted,” “Worried that they could not go home during the Spring Festival,” and “Expressed panic about the epidemic and hoped to work at home” were obtained. Topics with a positive sentiment tendency were “Hope the epidemic will end soon and the world will get well,” “Praise the government for effective control measures,” and “Salute medical workers and volunteers in communities.”

#### Comparison of the six cases

To answer the second research question—*Were there any differences regarding the social media content of RIDCCV between these six cases?*—we conducted a comparative analysis regarding the similarities and differences. To provide a more comprehensive understanding of public opinion, we divided the six cases into two groups based on whether the person violated epidemic prevention and control measures. Group 1 contained cases in which the subjects violated the regulations (RCase 2, RCase 5, and RCase 6), and Group 2 contained cases in which the subjects did not violate the regulations (RCase 1, RCase 3, and RCase 4).

On the one hand, the data reflected similarities, as follows:

- Some people expressed appreciation for and saluted the medical workers and the government. This finding is consistent with some previous studies focusing on social media data related to the COVID-19 pandemic in (Duan et al., 2021; Hou et al., 2021; Luo et al., 2021), indicating the public’s common approval of Chinese epidemic prevention policies.
- Opinions about fears concerning disease outbreaks appeared in the data of most researched cases. Many such opinions were generally found in social media data related to the COVID-19 pandemic, regardless of the research topic (Luo et al., 2021).
- The results of text mining for RCase 1, RCase 4, and RCase 6 showed that some people worried that they could not return home for the holidays. This opinion was expressed mainly because these three cases occurred around the Spring Festival and National Day rather than during the event itself.
- Regarding the sentiment analysis results, most opinions on social media expressed a negative sentiment. The proportion of posts with negative sentiment was 72.55%, 77.66%, 62.54%, 85.66%, 84.53%, and 73.33% in the six cases, respectively.

On the other hand, regarding the differences between the two groups, the focus of the negative opinions was very different. As shown in Fig. 2, in RCase 2, most negative opinions involved condemnations of the woman because she broke quarantine. Moreover, in RCase 6, many negative opinions expressed the person’s condemnation and claimed he should be heavily sentenced. However, in RCase 1, as displayed in Fig. 1, some negative opinions were directed at those engaging in CV against the woman and those spreading rumors and disclosing her private information. Notably, negative opinions about personal attacks on her private life were only expressed at the beginning of the Spring Festival, after which public opinions on social media gradually shifted from CV against the women to denouncing and condemning those who had divulged the private information and spread rumors with the development of the event.

In RCase 4, many negative opinions expressed the condemnation of CV behavior. For instance, a user posted, “Cyber violence should be punished. It has a great impact on people…” Nevertheless, it is evident that although the old couple in RCase 5 violated the epidemic prevention and control regulations, there were still some negative opinions expressing the condemnation of CV behavior, similar to the cases in Group 2. This is because the negligence of epidemic prevention workers in Jiayuguan was also a primary reason for the epidemic spreading in Xi’an. In addition, because the couple was older, many people could not bear seeing them suffer from CV.

**Fig. 7:**
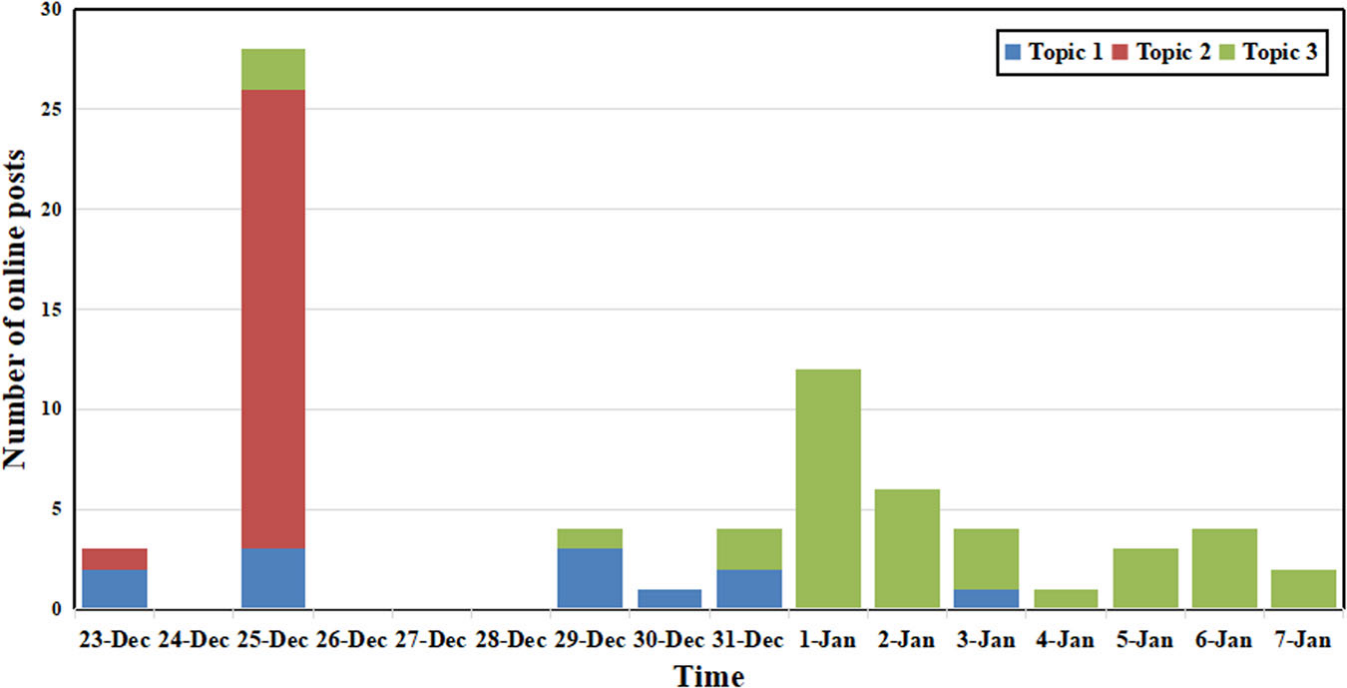
Changes of three main topics with time. This figure shows the changes in the proportion of posts related to the three topics over time.

Moreover, because RCase 1 and RCase 2 occurred at roughly the same time, there were some online posts containing discussions comparing the two cases, which may yield new findings. Thus, we extracted online posts containing at least one of the following keywords: “Chengdu,” “Pixian,” or “girl” from the data of RCase 2. Then, those posts focusing explicitly on a comparison between the two cases were manually selected—a total of 72 posts that included three main topics, as shown in Table 1. The three main topics were “Hope the epidemic will end soon and call on the government to find out the source of the epidemic as soon as possible,” “Call on the government to strengthen management in route information disclosure,” and “Sympathize with the girl in Case 1 and think the woman in RCase 1 deserves cyber violence and is not worth being pitied.” The changes in the proportion of posts related to the three topics over time are shown in Fig. 7. The figure shows that most people had opinions about Topic 2 and Topic 3 when comparing RCase 1 and RCase 2.

Specifically, in the early stage of RCase 1, Topic 1 was of greatest concern and peaked on December 25. This is mainly because the woman’s relatives posted on social media that their personal information had been leaked and that they had been harassed and attacked since December 23. They also added that they had actively reported personal information to the government as per government epidemic prevention regulations in Shenyang. However, the information leak subjected them to CV, which they had to endure in addition to COVID-19 infection. In the later stage of RCase 2, Topic 3 accounted for the largest proportion of posts, remaining a dominant issue until January 7, 2021. These findings support the argument that people commonly resisted CV against infected patients if they had not intentionally spread the virus to others and had not violated the regulations.

### Social network analysis

**Fig. 8:**
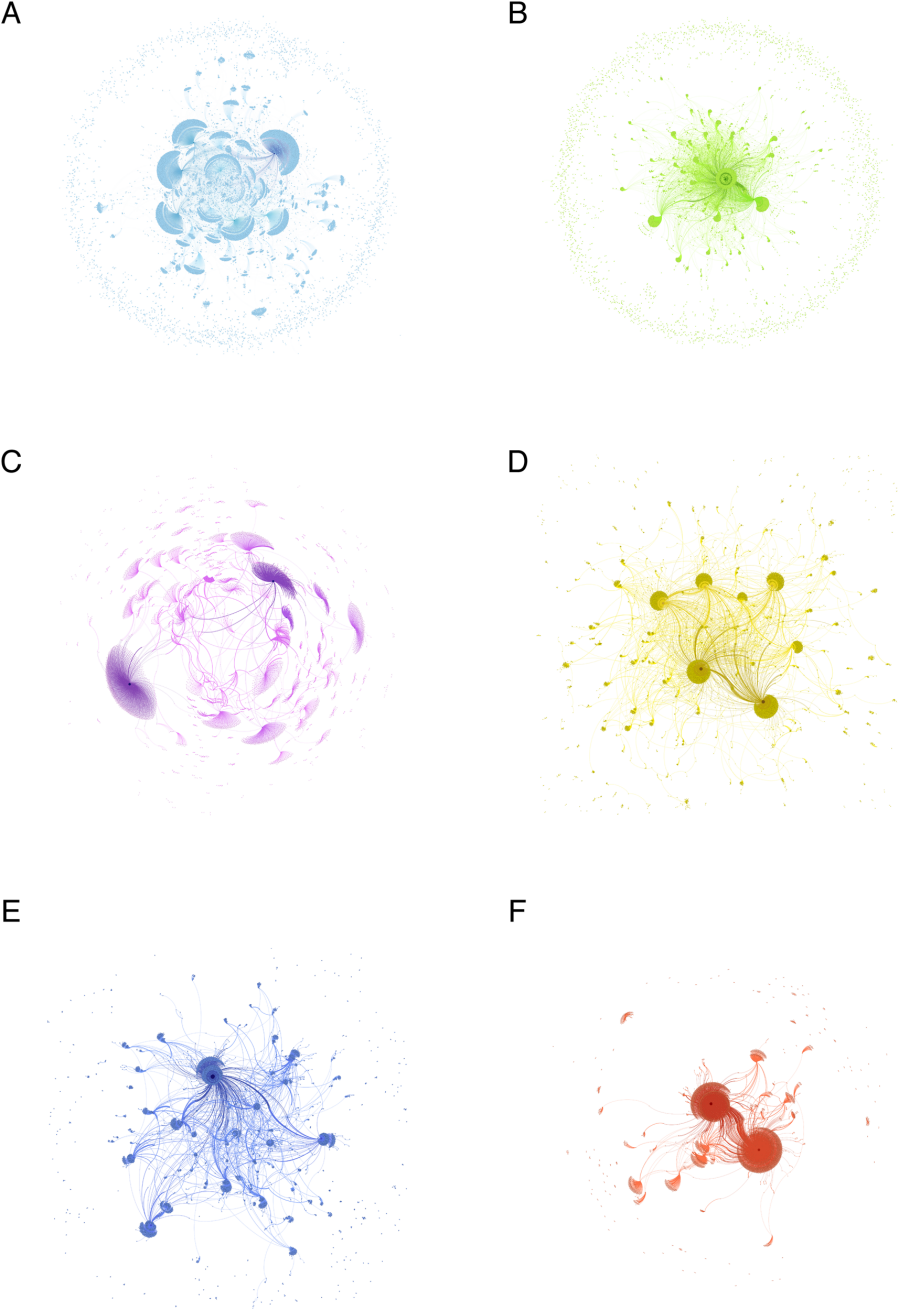
Spreading networks of six RCases. **A** Spreading network of RCase 1. **B** Spreading network of RCase 2. **C** Spreading network of RCase 3. **D** Spreading network of RCase 4. **E** Spreading network of RCase 5. **F** Spreading network of RCase 6.

To answer the third research question—*Who plays an important role in disseminating information on social media?—*we conducted a spreading network analysis. Using social network analysis, we constructed the network spread of the six cases based on the original posts and comments. Figure 8 displays these networks. We then employed the weighted PageRank algorithm proposed by Zhang (Zhang, 2017) to explore the importance of each user. This study set the parameter for controlling the algorithm’s performance at 0.85. Finally, based on the classification method demonstrated in section “Social network analysis”, the users contained in our constructed networks were divided into four groups: mainstream/official media, influential we-media, we-media, and everyday users. The top 10 nodes with the largest PageRank values of the six cases were selected and classified, as shown in Table 4. Most of the selected users were mainstream media or influential we-media users, accounting for 50% and 30% in RCase 1, 70% and 30% in RCase 2, 10% and 70% in RCase 3, 50% and 50% in RCase 4, and 80% and 20% in RCase 5 and RCase 6, respectively.

**Table 4:** Identified top 10-users with the largest PageRank values of six cases.

RCase 1				RCase 2				RCase 3			
Order	Name	PageRank	Type	Order	Name	PageRank	Type	Order	Name	PageRank	Type
1	央**闻	0.113784	M	1	人**报	0.071378	M	1	央**闻	0.00062	M
2	人**报	0.05513	M	2	央**闻	0.045957	M	2	丁**园	0.000558	I
3	大**文	0.035656	I	3	封**闻	0.041684	I	3	圈**姐	0.000464	I
4	中**网	0.01526	M	4	四**报	0.04042	M	4	丁**生	0.000449	I
5	辽**报	0.013922	M	5	成**布	0.032104	M	5	陈**体	0.000421	N
6	沈**布	0.011558	M	6	中**网	0.023655	M	6	我**事	0.00042	I
7	心**o	0.008558	W	7	四**察	0.017563	M	7	时**者	0.00042	I
8	妖**略	0.007743	I	8	孤**野	0.010581	I	8	黄**耶	0.000383	I
9	北**瑶	0.007583	N	9	四**布	0.009638	M	9	瑞**学	0.000382	N
10	澎**闻	0.007467	I	10	澎**闻	0.00941	I	10	沸**频	0.000374	I

RCase 4				RCase 5				RCase 6			
Order	Name	PageRank	Type	Order	Name	PageRank	Type	Order	Name	PageRank	Type
1	人**报	0.001558	M	1	北**报	0.000294	M	1	人**报	0.000372	M
2	新**报	0.000912	M	2	中**网	0.000285	M	2	央**闻	0.000372	M
3	新**闻	0.000713	M	3	人**报	0.000272	M	3	澎**闻	0.000371	I
4	央**闻	0.000694	M	4	紧**叫	0.000252	I	4	中**网	0.000354	M
5	西**策	0.000694	I	5	央**闻	0.00025	M	5	新**频	0.000354	M
6	凯**雷	0.000549	I	6	钱**频	0.000244	M	6	锦**闻	0.000354	M
7	紧**叫	0.000549	I	7	中**刊	0.000242	M	7	H**R	0.000354	I
8	央**看	0.000482	M	8	澎**闻	0.000223	I	8	最**都	0.000288	M
9	新**点	0.000464	I	9	鹤**察	0.000217	M	9	四**团	0.000284	M
10	周**良	0.000453	I	10	中**报	0.000214	M	10	环**报	0.000261	M

M measures the mainstream/official media; I measures the influential we media; W measures the non-influential we media; N measures the normal users.

## Discussion and conclusions

### Main findings

This study conducted a content analysis focusing on CV behavior caused by travel route information disclosure during the COVID-19 pandemic. First, 13 representative cases of RIDCCV that occurred between November 2020 and November 2021 in China were selected to identify common features. Then, six cases were analyzed and compared using public opinion data from social media and text mining approaches. This approach aimed to understand public attitudes toward RIDCCV on social media, the differences between the six cases, and how to effectively avoid RIDCCV during future pandemics and other emergencies. Based on the results, the answers to the research questions posed are as follows:

1. For the first question, we found that the Chinese public generally agrees with the current strict epidemic prevention policies implemented by the Chinese government but has also experienced anxiety due to the restrictions imposed on travel, especially during holidays, such as the Spring Festival and National Day. For the six analyzed cases, many opinions condemning the infected patients for their actions, which spread the COVID-19 virus, can be found on social media, some of which expressed CV. In addition, some people also held opinions against CV against the patients, especially the girls in RCase 1 and RCase 3, as they believed that the persons were victims and did not spread COVID-19 on purpose. Moreover, many people called on the government to strictly protect patients’ privacy and establish an effective and reasonable route information disclosure mechanism.
2. For the second question, we found that although most people expressed negative sentiments, the focus of their negative opinions in the six cases was substantially different. In particular, people resisted CV against patients if they had not intentionally spread the virus to others without breaking the law. This finding offers a more detailed understanding of the formation mechanism of CV behavior during emergencies—intentional harm is the root cause of CV.
3. For the third question, we created six spreading networks based on the original posts and comments of six cases. A weighted PageRank analysis was conducted to explore the importance of users in the networks. We found that mainstream media and influential we-media played an essential role.

We concluded that publishing the route information of infected patients can assist the public in being better aware of their proximity to patients so that they can effectively protect themselves and thereby reduce their anxiety. To date, to a large extent, the COVID-19 pandemic has been effectively controlled in some countries, such as China. However, there are still many areas where COVID-19 remains a serious and complex threat. Thus, the publication of route information of infected patients cannot be stopped for an extended period.

Nevertheless, there is no doubt that every individual has the right to privacy. Network technology provides people with a convenient and virtual communication platform to express their viewpoints. While enjoying this right, we must realize that we should be responsible for our words and deeds. It is a fact that some patients have expanded the range of infection due to a lack of awareness of prevention and control regulations. Their actions have burdened the public and society at large. However, we cannot use this as an excuse to engage in unwarranted and malicious speculation about patients; doing so may eventually evolve into slander, abuse, insults, and even violence against patients. As to whether patients should be held responsible for the spread of the pandemic or criminally investigated for their actions, these are the responsibilities of government authorities, not individual citizens.

### Formation mechanism of RIDCCV in the COVID-19 pandemic

This sub-section explains the formation mechanism of RIDCCV during the pandemic. From the perspective of motivation, all of our considered RIDCCV cases could be classified as the same kind of CV event. They were all related to ethically sensitive social “hot topics” during a particular period and originated with an initial call for justice. People expected each other to uphold epidemic prevention and control regulations and sought to punish those who violated these measures by posting opinions on social media (Liao et al., 2020). People approach the arena of virtual opinions as a highly liberalized, open, and anonymous cyberplace, but also as a platform for social supervision and catharsis concerning negative emotions. During the COVID-19 pandemic, when the published route information showed that someone had violated epidemic prevention regulations, people were quick to reach a consensus and establish a new set of moral norms based on a traditional public order to swiftly render a moral judgment of violators on the Internet, which ultimately evolved into RIDCCV.

For example, “three kinds of obedience and four virtues” is the primary embodiment of traditional Chinese moral values of women. Specifically, three kinds of obedience include daughters to their fathers before marriage, wives to their husbands after marriage, and widows to their sons after their husband’s death. The four virtues include morality, proper speech, modest manner, and diligent work (Basu et al., 2017). Although this standard has been widely criticized for a long time, its influence on today’s moral foundations in China is still significant (Zuo et al., 2012). Currently, women are expected to always be modest in manner and kept passive and sexually innocent in their relationships with men (Zuo et al., 2012). These moral standards for women were widely discussed in RCase 1, calling into question the girl’s private life and purity. On a larger scale, leaked private information and exposed route information have served as the basis for moral trials of netizens, ultimately escalating into verbal abuse and other violent acts in the tense and panicked atmosphere of the COVID-19 pandemic. In addition, the characteristics of the cases displayed in Table 1 indicate that those who were identified as the first locally confirmed cases of a particular wave of the pandemic were more likely to be victims of CV.

Rumors are also the primary cause of RIDCCV on social media during emergencies (Barlett et al., 2021). In some cases, rumors about patients and their families were quickly generated after the government exposed the route information of infected people. These rumors became a justification for people to use offensive language and launch personal attacks, which finally escalated into CV. A previous study found that distorted and exaggerated rumors generally spread rapidly in public emergencies (Lu et al., 2020). Social contradictions and negative emotions in the rumors stimulate and incite the corresponding moral principles of people. According to previous studies, uncertainty, and disturbed environment, as well as the degree of relevance to the public, are positively correlated with rumor propagation (Kim, 2018). The occurrence of public emergencies increases the uncertainty of the information environment. In contrast, fears about disease and death increase the uneasiness of the audience, especially in areas with high-security risks, and the technology of social media increases the relevance of rumors to the audience. Therefore, rumors spread more widely in the context of public health emergencies and are more readily accepted by the audience, culminating in the occurrence of CV.

Moreover, group polarization plays an influential role in RIDCCV (Bertolotti et al., 2013). According to existing research, online public opinions concerning sudden, unexpected public events have the characteristics of information flooding and uncertainty, which immerse people in a panicked atmosphere (Keller et al., 2021) and consequently increase the probability and degree of group polarization. In the six cases, after the route information of patients had been exposed, the behavioral motivation of CV evolved from “justice” to purely malicious attacks to achieve emotional catharsis. A large number of public opinions about dissatisfaction with adverse effects in daily life and work were posted on social media, which resonated with many netizens in unaffected areas. Finally, under the influence of an irrational and extreme emotional atmosphere, public opinions tend to be consistent, thereby enabling the group polarization inherent in CV to form.

### Governance measures for RIDCCV

Based on our study findings, we recommend several measures to prevent RIDCCV during emergencies, such as the COVID-19 pandemic.

#### Optimize the route information collection and disclosure system

In most cases, grassroots staff in government agencies are in charge of publishing the necessary personal information about confirmed patients and their close contacts. These staff are the most likely to cause information leakage. Therefore, the government should balance the requirements for epidemic prevention and protecting people’s privacy by formulating and optimizing personal route information collection and disclosure mechanisms. In addition, more extensive training is also needed to enhance privacy protection awareness and legal literacy among grassroots staff. Since the beginning of 2021, Beijing and Shanghai have adopted the epidemiological survey reporting method of “only mentioning the route but not people,”^1^ which no longer discloses the age, gender, and other personal information of confirmed cases. This method not only achieves the purpose of disseminating epidemic information, but also ensures the protection of patients’ personal information and reduces the risk of privacy disclosure. This strategy is, therefore, worth advocating in cities around the world. In addition, the government should pay more attention to patients who are the first locally confirmed cases of a specific wave of an epidemic or pandemic, as they are more likely to be victimized by CV on the Internet.

#### Ease public anxiety about the COVID-19 pandemic

Changeable social interests, an open network environment, and the complex psychology of netizens partly accelerate the formation of CV (Jain et al., 2020). On one hand, it is necessary to purify the network communication ecology from top to bottom and standardize the governance of cyberspace. On the other hand, during public health emergencies, such as the COVID-19 pandemic, the psychological interaction between an uncertain environment and anxiety has become the breeding ground for CV. In addition, social emotions generally become more volatile and uncertain when public health emergencies occur (Li et al., 2022). Thus, the government also needs to confront the negative emotions of the public, such as fear and dissatisfaction, by understanding and addressing their most pressing problems and grasping the psychological characteristics of people in cyberspace from the bottom to the top. In particular, service teams composed of mental health professionals and social workers should be established to provide psychological services for cured and isolated patients and ordinary people who think they have psychological problems because of the pandemic. The focus of these services should include psychological counseling for people with anxiety, depression, insomnia, and post-traumatic stress disorder (Redmond et al., 2020). Moreover, the government must promptly identify people prone to self-injury, suicide, attacks, or other psychotic symptoms.

#### Promote publicity with the help of mainstream media and influential we-media

When emergencies occur, the mainstream media and influential we-media focus on reporting hot events, which may arouse widespread attention from the government and the public. In most cases, the more emphasis is given by the mainstream media, and the more influential we become, the higher the public’s attention to an issue. According to the agenda-setting theory, the agenda-setting function of mainstream media and influential we-media offers a deeper understanding of discussed topics; the media agenda affects the public agenda (Liu et al., 2011). In other words, the mainstream media and influential we-media play an essential role as opinion leaders in guiding public opinion on the Internet (Lai et al., 2021). Therefore, the government should encourage the mainstream media and influential we-media to persistently report facts, from the announcement of official epidemic prevention and control regulations to people’s personal protection actions and shift the public’s attention from private issues to public issues to maintain and promote the publicity of the media. In addition, mainstream media and influential we-media should be responsible for preventing the generation and spread of rumors.

In terms of CV, mainstream media and influential we-media should take full advantage of their capacities, such as large audiences and the authority to act as gatekeepers of information transmission during emergencies. When CV appears on the Internet, mainstream media and influential we-media should increase the number and strengthen the intensity and depth of reports about the incidents that triggered CV to enable people to better judge the authenticity of the information and guide them toward a deeper understanding of the actual situation and the true meaning of the incidents.

### Implications of this study

The implications of this study are as follows:

1. This study identified a crucial overlapping area in the social media, communication, and crisis management literature, specifically the intersection between CV and epidemic prevention. In addition, the study was one of the first to assess CV behavior on social media as caused by route information disclosure during the COVID-19 pandemic. Our findings indicate that, for RIDCCV, people are naturally resistant to CV against patients who may have unknowingly spread the virus to others without breaching the law. In addition, some characteristics that commonly appeared in RIDCCV were identified, such as the existence of rumors and moral condemnation caused by the official route information disclosure, and that those patients who are the first locally confirmed cases of a particular wave of the pandemic are more likely to be victimized by CV.
2. This study conducted content and network analyses focusing on social media data related to six RIDCCV cases in China. The formation mechanism of RIDCCV was proposed. As information disclosure is one of the commonly applied countermeasures in emergencies, these findings could be applied to network governance strategies for government departments during other major emergencies, such as natural disasters, accident disasters, and national security events. Therefore, our study enhances network governance and crisis management knowledge.
3. This study highlighted the importance and necessity of privacy protection in route information disclosure during the COVID-19 pandemic. We believe that information disclosure can effectively alleviate public panic and bolster trust in the government. However, effective control of virus transmission cannot be realized at the expense of patient privacy.
4. Based on our findings, we offer three practical recommendations:
  - Promote the publicity of the media field with the help of mainstream media and influential we-media.
  - Optimize the route information collection and disclosure system.
  - Ease public anxiety about the COVID-19 pandemic.

These recommendations are of great practical significance for the government to better balance the route information disclosure of patients to prevent CV during global emergencies. Government institutions can use social media to respond to public requests in a comprehensive and timely manner. This could also promote excellent two-way communication during critical crisis moments. Such actions would help preserve and develop the relationship between the government and the general public.

### Study limitations and future research directions

This study had several limitations that future studies could address.

1. The effects of different patient characteristics, such as gender, age, and occupation, on CV were not considered. In future studies, we will propose a more detailed analysis of CV for a variety of patient types identified during the COVID-19 pandemic.
2. The six considered cases occurred during one specific period of the pandemic in China. However, we believe that the public’s focus shifts in stages during crises, generating different factors that could, in turn, lead to CV. Thus, future research will consider more RIDCCV cases in different periods of the COVID-19 pandemic.
3. Other factors, such as the types of government departments and stages of crises that influence the governance of CV, also warrant further research. For example, CV governance is a complex task during the crisis, requiring the participation and collaboration of many government departments, including but not limited to communications, education, and police. Thus, it is worth studying how to effectively combine these departments and build a collaborative CV governance system. In addition, the early stage of a crisis should receive significant attention in future research, as governance measures are generally incomplete and imperfect. Those imperfections may lead to a higher risk of privacy leaks and subsequent CV.

All of these issues should be considered in future studies.

## Supplementary information

SUPPLEMENTAL MATERIAL

## Data Availability

The datasets generated during and/or analyzed during the current study are available from the corresponding author on reasonable request. The codes used in this article are included in the Supplementary Information.

## Footnotes

1 https://baijiahao.baidu.com/s?id=1689753315143554756&wfr=spider&for=pc.
