# Supplementary material for: Cyber violence caused by the disclosure of route information during the COVID-19 pandemic
Source: Humanit Soc Sci Commun. 2022 Nov 25;9(1):417. doi: 10.1057/s41599-022-01450-8 (PMC9702928; doi:10.1057/s41599-022-01450-8)
Supplement: Supplementary file 1 — SUPPLEMENTAL MATERIAL [file 41599_2022_1450_MOESM1_ESM.docx]

**Supplemental Information**

Route Information Disclosure-Caused Cyber Violence in COVID-19 Pandemic: A Case Study Based on Text Mining and Social Network Analysis

**1. Codes of Bi-LSTM for sentiment classification (Python)**

import torch

import torch.nn as nn

import torch.nn.functional as F

from torch.autograd import Variable

torch.manual_seed(123)

class BLSTM(nn.Module):

def __init__(self, embeddings, input_dim, hidden_dim, num_layers, output_dim, max_len=40, dropout=0.5):

super(BLSTM, self).__init__()

self.emb = nn.Embedding(num_embeddings=embeddings.size(0),

embedding_dim=embeddings.size(1),

padding_idx=0)

self.emb.weight = nn.Parameter(embeddings)

self.input_dim = input_dim

self.hidden_dim = hidden_dim

self.output_dim = output_dim

self.sen_len = max_len

self.sen_rnn = nn.LSTM(input_size=input_dim,

hidden_size=hidden_dim,

num_layers=num_layers,

dropout=dropout,

batch_first=True,

bidirectional=True)

self.output = nn.Linear(2 * self.hidden_dim, output_dim)

def bi_fetch(self, rnn_outs, seq_lengths, batch_size, max_len):

rnn_outs = rnn_outs.view(batch_size, max_len, 2, -1)

fw_out = torch.index_select(rnn_outs, 2, Variable(torch.LongTensor([0])).cuda())

fw_out = fw_out.view(batch_size * max_len, -1)

bw_out = torch.index_select(rnn_outs, 2, Variable(torch.LongTensor([1])).cuda())

bw_out = bw_out.view(batch_size * max_len, -1)

batch_range = Variable(torch.LongTensor(range(batch_size))).cuda() * max_len

batch_zeros = Variable(torch.zeros(batch_size).long()).cuda()

fw_index = batch_range + seq_lengths.view(batch_size) - 1

fw_out = torch.index_select(fw_out, 0, fw_index) # (batch_size, hid)

bw_index = batch_range + batch_zeros

bw_out = torch.index_select(bw_out, 0, bw_index)

outs = torch.cat([fw_out, bw_out], dim=1)

return outs

def forward(self, sen_batch, sen_lengths, sen_mask_matrix):

sen_batch = self.emb(sen_batch)

batch_size = len(sen_batch)

sen_outs, _ = self.sen_rnn(sen_batch.view(batch_size, -1, self.input_dim))

sen_rnn = sen_outs.contiguous().view(batch_size, -1, 2 * self.hidden_dim) # (batch, sen_len, 2*hid)

sentence_batch = self.bi_fetch(sen_rnn, sen_lengths, batch_size, self.sen_len) # (batch_size, 2*hid)

representation = sentence_batch

out = self.output(representation)

out_prob = F.softmax(out.view(batch_size, -1))

return out_prob

def train(model, training_data, args, optimizer, criterion):

model.train()

batch_size = args.batch_size

sentences, sentences_seqlen, sentences_mask, labels = training_data

assert batch_size == len(sentences) == len(labels)

sentences_, sentences_seqlen_, sentences_mask_ = \

var_batch(args, batch_size, sentences, sentences_seqlen, sentences_mask)

labels_ = Variable(torch.LongTensor(labels))

if args.cuda:

labels_ = labels_.cuda()

assert len(sentences) == len(labels)

model.zero_grad()

probs = model(sentences_, sentences_seqlen_, sentences_mask_)

loss = criterion(probs.view(len(labels_), -1), labels_)

loss.backward()

optimizer.step()

model.zero_grad()

probs = model(sentences_, sentences_seqlen_, sentences_mask_)

loss = criterion(probs.view(len(labels_), -1), labels_)

loss.backward()

optimizer.step()

def test(model, dataset, args, data_part="test"):

tvt_set = dataset[data_part]

tvt_set = yutils.YDataset(tvt_set["xIndexes"],

tvt_set["yLabels"],

to_pad=True, max_len=args.sen_max_len)

test_set = tvt_set

sentences, sentences_seqlen, sentences_mask, labels = test_set.next_batch(len(test_set))

assert len(test_set) == len(sentences) == len(labels)

tic = time.time()

model.eval()

batch_size = len(sentences)

sentences_, sentences_seqlen_, sentences_mask_ = \

var_batch(args, batch_size, sentences, sentences_seqlen, sentences_mask)

probs = model(sentences_, sentences_seqlen_, sentences_mask_)

_, pred = torch.max(probs, dim=1)

if args.cuda:

pred = pred.view(-1).cpu().data.numpy()

else:

pred = pred.view(-1).data.numpy()

tit = time.time() - tic

labels = numpy.asarray(labels)

return labels

**2. Codes of LDA (R)**

library(NLP)

library(topicmodels)

LDA<-function(dtm){

unique_lines = unique(dtm$i)

dtm = dtm[unique_lines,]

Gibbs = LDA(dtm, k =6, method = "Gibbs",control = list(seed = 2015, burnin = 1000,thin = 100, iter = 1000))

Topic1 <- topics(Gibbs, 1)

table(Topic1)

Terms1 <- terms(Gibbs, 25)

Terms1

}

**3. Codes of TF-IDF (R)**

library(NLP)

library(topicmodels)

TFI<-function(x){

keys = worker("keywords",topn=200,idf=IDFPATH)

oo<-vector_keywords(x,keys)

print(oo)

}
